# Effectiveness of injectable risperidone long-acting therapy for schizophrenia: data from the US, Spain, Australia, and Belgium

**DOI:** 10.1186/1744-859X-10-10

**Published:** 2011-04-04

**Authors:** Tim Lambert, José M Olivares, Joseph Peuskens, Cherilyn DeSouza, Chris M Kozma, Patrick Otten, Concetta Crivera, An Jacobs, Wayne Macfadden, Lian Mao, Stephen C Rodriguez, Riad Dirani, Kasem S Akhras

**Affiliations:** 1 University of Sydney, Sydney, Australia; 2 Hospital Meixoeiro, Complejo Hospitalario Universitario de Vigo, Vigo, Spain; 3 Universitair Psychiatrisch Centrum, KU Leuven Campus UC-St. Jozef, Kortenberg, Belgium; 4 Veterans Affairs Medical Center, Kansas City, MO, USA; 5 University of South Carolina, Columbia, SC, USA; 6 SGS Life Science Services, Mechelen, Belgium; 7 Ortho-McNeil Janssen Scientific Affairs, LLC, Titusville, NJ, USA; 8 Johnson & Johnson Pharmaceutical Services, Beerse, Belgium; 9 Formerly Ortho-McNeil Janssen Scientific Affairs, LLC, Titusville, NJ, USA; 10 Johnson & Johnson Pharmaceutical Research and Development, LLC, Titusville, NJ, USA; 11 Johnson & Johnson Pharmaceutical Services, Raritan, NJ, USA

## Abstract

**Background:**

Because wide variations in mental health care utilization exist throughout the world, determining long-term effectiveness of psychotropic medications in a real-world setting would be beneficial to physicians and patients. The purpose of this analysis was to describe the effectiveness of injectable risperidone long-acting therapy (RLAT) for schizophrenia across countries.

**Methods:**

This was a pragmatic analysis of data from two prospective observational studies conducted in the US (Schizophrenia Outcomes Utilization Relapse and Clinical Evaluation [SOURCE]; ClinicalTrials.gov registration number for the SOURCE study: NCT00246194) and Spain, Australia, and Belgium (electronic Schizophrenia Treatment Adherence Registry [eSTAR]). Two separate analyses were performed to assess clinical improvement during the study and estimate psychiatric hospitalization rates before and after RLAT initiation. Clinical improvement was evaluated using the Clinical Global Impressions-Severity (CGI-S) and Global Assessment of Functioning (GAF) scales, and change from baseline was evaluated using paired *t *tests. Psychiatric hospitalization rates were analyzed using incidence densities, and the bootstrap resampling method was used to examine differences between the pre-baseline and post-baseline periods.

**Results:**

The initial sample comprised 3,069 patients (US, n = 532; Spain, n = 1,345; Australia, n = 784; and Belgium, n = 408). In all, 24 months of study participation, completed by 39.3% (n = 209), 62.7% (n = 843), 45.8% (n = 359), and 64.2% (n = 262) of patients from the US, Spain, Australia, and Belgium, respectively, were included in the clinical analysis. Improvements compared with baseline were observed on both clinical assessments across countries (*P *< 0.001 at all post-baseline visits). The mean improvement was approximately 1 point on the CGI-S and 15 points on the GAF. A total of 435 (81.8%), 1,339 (99.6%), 734 (93.6%), and 393 (96.3%) patients from the US, Spain, Australia, and Belgium, respectively, had ≥1 post-baseline visit and were included in the analysis of psychiatric hospitalization rates. Hospitalization rates decreased significantly in all countries regardless of hospitalization status at RLAT initiation (*P *< 0.0001) and decreased significantly in the US and Spain (*P *< 0.0001) when the analysis was limited to outpatients only.

**Conclusions:**

RLAT in patients with schizophrenia was associated with improvements in clinical and functional outcomes and decreased hospitalization rates in the US, Spain, Australia, and Belgium, despite differences in health care delivery systems.

## Background

According to analyses by the World Health Organization (WHO), wide variations exist in mental health care delivery systems across the world; for example, only 68% of countries have a mental health care policy [1]. Countries differ with respect to the primary method of financing mental health care (that is, out-of-pocket payment, tax-based, social insurance, private insurance, or external grants) and available funds allocated for mental health care [1]. Schizophrenia is a particularly debilitating mental illness with high human and societal costs. Patients and their families cope with symptom fluctuations, poor social and occupational functioning, and the periodic need for psychiatric hospitalization due to relapse [2,3]. For society, schizophrenia is one of the most expensive mental illnesses to treat, with psychiatric hospitalization being a key driver of costs [4].

Continuous antipsychotic therapy is recommended to limit disease severity. However, with oral antipsychotic medication, non-adherence is present in approximately half of patients [5]. Partial adherence (taking some but not all medication as prescribed) is even more prevalent, occurring in approximately 9 out of 10 patients [6]. The impact of poor medication adherence is substantial and includes increased symptom levels; a greater risk for violence, arrest, and poor functioning; higher rates of substance abuse and alcohol-related problems; increased risk of psychiatric hospitalization and increased hospital costs [6-10]. A recent (2005) estimate of US costs due to medication non-adherence in patients with schizophrenia was $1.479 billion [11]. Additionally, each relapse episode predisposes the patient to further episodes, whereas fewer episodes are associated with better long-term outcomes [12].

Long-acting formulations can improve adherence by ensuring medication delivery between injections. Compared with oral medications, depot formulations offer better control over dose adjustments, more predictable and consistent plasma drug concentrations, and lower rates of patient relapse [13,14]. Risperidone long-acting therapy (RLAT) is the first such treatment to combine a long-acting injectable formulation with the efficacy of a second-generation antipsychotic. The short-term and long-term efficacy and safety of RLAT in patients with schizophrenia have been demonstrated in randomized controlled studies [15-18], and one open-label study (N = 397) found that the annual rehospitalization rate decreased from 38% to 12% in those receiving RLAT (*P *< 0.001) [19].

The objective of this pragmatic analysis was to examine the long-term effectiveness of RLAT in real-world clinical practice in different countries. Data for this analysis were from two 2-year RLAT observational studies: the Schizophrenia Outcomes Utilization Relapse and Clinical Evaluation (SOURCE) study, conducted in the US, and the electronic Schizophrenia Treatment Adherence Registry (eSTAR), conducted in Spain, Australia, and Belgium.

The health care systems in these countries vary considerably. The US spends 6% of its total health budget on mental health care [1]. The primary sources of financing are private insurance, tax-based insurance, and out-of-pocket expenditures paid by the patient or family. When private insurance benefits are exhausted, patients move to the public sector, where Medicaid and Social Security Disability Insurance provide a safety net [1]. Spain does not have a specific budget allocation for mental health care services, so details about mental health care expenditures in that country are not available. The Spanish health care system provides universal access to medical and mental health care services for all of its citizens [1]. Australia has a national health care system with universal access for all citizens. It spends 10% of its total health budget on mental health care, and the primary source of mental health care financing is tax based [1]. Belgium spends 6% of its total health budget on mental health care services, which are a part of the primary health care system. The primary source of mental health care financing is through social insurance [1].

## Methods

### SOURCE and eSTAR designs

Data from the two studies were collected under similar protocols, which imposed no study-mandated interventions except for initiation of RLAT. The SOURCE study (CR005035) was conducted from September 2004 to October 2007 in community mental health centers and Veterans Affairs Medical Centers in the US. In eSTAR (CR005548), a multinational study conducted in 17 countries, participating physicians enrolled patients after treatment was initiated with RLAT during the patient's routine clinical management. Patients were enrolled in eSTAR between December 2003 and July 2009. Because Spain, Belgium, and Australia completed 24 months of patient treatment, the data from these three countries only were used for this analysis. Institutional review boards or ethics committees, as appropriate, provided approval of the respective study protocols.

### Participants

Participants were male or female patients with schizophrenia, aged 18 years or older, who required initiation of RLAT as determined by their physicians. Pregnant or breastfeeding patients and patients considered treatment resistant were excluded. All patients (or their legal representatives) provided written informed consent before participation in the studies.

### Treatment

The recommended RLAT dose is 25 mg administered every 2 weeks. The initial dose chosen for each patient, however, was based on the individual physician's judgment. Any subsequent decisions regarding treatment (including stopping, starting, or changing RLAT and prescribing concomitant psychiatric medications) were made as deemed appropriate by the treating physician.

### Data collection

Retrospective data on psychiatric hospitalizations were determined for the 12-month period before enrollment. At baseline (initial dose of RLAT), demographic information was collected and the Clinical Global Impressions-Severity (CGI-S) [20] and Global Assessment of Functioning (GAF) [21] were assessed. The CGI-S is a seven-point scale ranging from 1 (normal, not at all ill) to 7 (severely ill). The GAF is a single-item rating of the patient's psychological, social, and occupational functioning, with scores ranging from 0 to 100 and higher scores indicating better functioning. Hospitalization rates and CGI-S and GAF scores were determined at 3-month intervals after the baseline visit, up to month 24.

### Statistical methods

During the study, CGI-S and GAF scores were reported for all patients who completed 24 months of treatment. Change from baseline in CGI-S and GAF scores was evaluated using paired *t *tests. All tests were two tailed and conducted at the 5% significance level. No adjustments were made for multiplicity.

Data were analyzed for each country independently. Psychiatric hospitalization rates were analyzed using incidence densities, defined as the total number of events for the study population divided by the total length of treatment in years. Patients who had only a baseline visit with no treatment information were excluded from the analysis. This analysis included 12 months of pre-baseline data and 24 months of post-baseline data, adjusted for 1 year. The bootstrap resampling method was used to calculate 95% confidence intervals to examine differences between the pre-baseline and post-baseline periods. To account for the differences between countries in the percentage of patients hospitalized at baseline, incidence ratios were calculated for (1) all patients regardless of hospitalization status and (2) outpatients. For patients who were hospitalized when the initial dose of RLAT was administered, the current hospitalization was considered as occurring before baseline.

## Results

### Patients

Baseline demographic and clinical characteristics for the patients who completed 24 months of treatment are included in Table 1. In all, 24 months of treatment were completed by 39.3% of patients in the US, 62.7% in Spain, 45.8% in Australia, and 64.2% in Belgium. Approximately two-thirds of the patients in all countries were male. The mean age ranged from 38.8 years (Spain) to 42.4 years (US). Noteworthy differences were observed in disease duration, with the mean time since diagnosis ranging from 10.6 years (Belgium) to 18.1 years (US). Baseline mean CGI-S scores were similar in all countries and indicated patients were moderately to markedly ill. Baseline mean GAF scores were slightly higher in the US and Spain than in Belgium and Australia, but patients in all countries had moderate to severe functional impairment (mean scores ranged from 42.3 to 48.3) (Table 1).

**Table 1:** Demographic and clinical characteristics at baseline

	US	Spain	Australia	Belgium
Patients, n	532	1,345	784	408

Patients who completed 24 months of treatment (evaluated for clinical measures)				

Patients with 24 months of follow-up, n (%)^a^	209 (39.3)	843 (62.7)	359 (45.8)	262 (64.2)

Age in years, mean (SD)	42.4 (12.3)	38.8 (11.2)	39.3 (13.0)	41.3 (13.3)

Male gender, %	64.1	63.2	68.5	63.7

Years since diagnosis, mean (SD)	18.1 (11.3)	13.1 (9.8)	12.2 (10.4)	10.6 (10.6)

CGI-S score, mean (SD)	4.6 (1.3)	4.6 (0.9)	4.5 (1.0)	4.7 (1.0)

GAF score, mean (SD)	48.3 (14.6)	47.8 (15.6)	42.3 (14.5)	43.3 (12.0)

Patients with ≥1 post-baseline visit (evaluated for psychiatric hospitalization rate)				

Patients with ≥1 post-baseline visit, n (%)^a^	435 (81.8)	1,339 (99.6)	734 (93.6)	393 (96.3)

Age in years, mean (SD)	41.9 (12.6)	38.4 (11.2)	37.1 (12.5)	40.3 (13.3)

Male gender, %	66.7	63.6	69.9	62.8

Years since diagnosis, mean (SD)	17.6 (12.1)	12.6 (9.5)	10.7 (9.5)	9.5 (10.2)

Hospitalized in the year before RLAT initiation, %	39.3	35.0	76.8	71.2

CGI-S score, mean (SD)	3.9 (1.2)	4.6 (0.9)	4.6 (1.0)	4.7 (1.0)

GAF score, mean (SD)	53.1 (13.7)	46.9 (15.2)	42.7 (14.4)	43.6 (12.5)

^a^Based on total patient population.

CGI-S = Clinical Global Impressions—Severity; GAF = Global Assessment of Functioning; RLAT = risperidone long-acting therapy.

The initial dose of RLAT varied considerably from country to country. RLAT 25 mg was the most common initial dose, used in 75.4% of all patients in the US, 43.2% in Spain, 90.8% in Australia, and 49.0% in Belgium. At the end of the study, the general trend was toward higher doses. The final dose, based on all patients in the study at 24 months, was 25 mg in 20% to 35% of all patients and 50 mg in 40.2% of US patients, 43.5% of Spanish patients, 36.2% of Australian patients, and 37.7% of Belgian patients.

### Change from baseline on CGI-S and GAF scores

**Figure 1:**
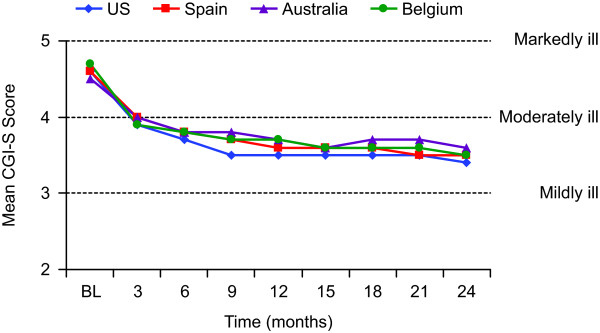
**Mean Clinical Global Impressions—Severity (CGI-S) scores by visit and country for patients who had 24 months of treatment**. All changes from baseline, *P *< 0.001, paired *t *test. BL = baseline.

Mean (SD) CGI-S scores at baseline were 4.6 (1.3), 4.6 (0.9), 4.5 (1.0), and 4.7 (1.0) in patients from the US, Spain, Australia, and Belgium, respectively. Mean CGI-S scores improved significantly (*P *< 0.001) in every country at all post-baseline visits up to 24 months. Changes were similar for patients regardless of country (Figure 1); mean scores decreased (improved) by approximately 0.6 to 0.8 points at 3 months and by 0.9 to 1.2 points at 24 months.

**Figure 2:**
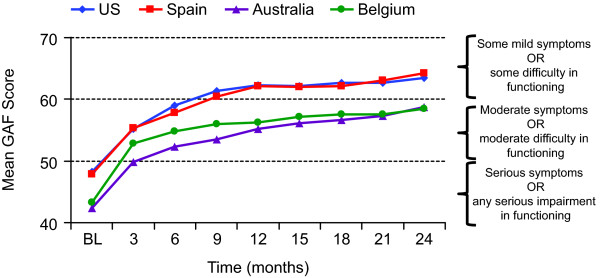
**Mean Global Assessment of Functioning (GAF) scores by visit and country for patients who had 24 months of treatment**. All changes from baseline, *P *< 0.001, paired *t *test. BL = baseline.

Mean (SD) GAF scores at baseline were 48.3 (14.6), 47.8 (15.6), 42.3 (14.5) and 43.3 (12.0) in patients from the US, Spain, Australia, and Belgium, respectively. GAF scores improved in each group over time; changes from baseline were statistically significant (*P *< 0.001) at all post-baseline visits up to 24 months (Figure 2). Across countries, scores improved by 6.9 to 9.6 points at 3 months and by 15.2 to 16.4 points at 24 months (Figure 2).

### Psychiatric hospitalization

Demographic and clinical characteristics at baseline for patients who had at least 1 post-baseline visit and were evaluated for hospitalization were similar to those of patients who completed 24 months of treatment (Table 1). The percentage of patients hospitalized in the year before RLAT initiation differed considerably, with the highest rates in Australia (76.8%) and Belgium (71.2%) and the lowest rates in the US (39.3%) and Spain (35.0%).

Psychiatric hospitalization was assessed in two groups of patients: (1) all patients regardless of hospitalization status at baseline and (2) outpatients (Table 2). The analysis of outpatients was performed to assess similar patient populations in the four countries, given the large between-country differences in the proportion hospitalized at baseline. The number of hospitalizations per person-year decreased significantly (*P *< 0.0001) in all countries when patients were considered regardless of baseline hospitalization status, with a percentage decrease of 54.9% in the US, 64.4% in Spain, 46.3% in Australia, and 52.4% in Belgium. When the analysis was limited to outpatients only, the number of hospitalizations decreased significantly for patients from the US and Spain, by 55.9% and 56.8%, respectively. The percentage decreases were 17.1% in Australia and 22.4% in Belgium (Table 2).

**Table 2:** Psychiatric hospitalizations 12 months before RLAT initiation for patients with ≥1 post-baseline visits (incidence density ratios)

Hospitalization status at baseline	US		Spain		Australia		Belgium	
	
	**All patients**^**a**^	Patients not hospitalized at baseline	**All patients**^**a**^	Patients not hospitalized at baseline	**All patients**^**a**^	Patients not hospitalized at baseline	**All patients**^**a**^	Patients not hospitalized at baseline
Patients, n	435	413	1,339	1,220	734	354	393	173

Before baseline visit	0.63	0.59	0.45	0.37	1.34	0.82	1.05	0.49

Change (95% CI)^b^	-0.35^c^ (-0.44 to -0.26)	-0.33^c^ (-0.42 to -0.24)	-0.29^c^ (-0.33 to -0.24)	-0.21^c^ (-0.25 to -0.17)	-0.62^c^ (-0.74 to -0.50)	-0.14 (-0.29 to 0.04)	-0.55^c ^ (-0.67 to -0.44)	-0.11 (-0.26 to 0.03)

Percentage change	-55.6%	-55.9%	-64.4%	-56.8%	-46.3%	-17.1%	-52.4%	-22.4%

Patients had data for the post-baseline period up to the time of study discontinuation. Estimates for the pre-baseline and post-baseline periods may be based on different observation time periods within patients. Hospitalizations occurring before study start or after the last study visit were not included in the analysis.

^a^For patients who were hospitalized when initiating RLAT, the current hospitalization was considered as occurring before baseline.

^b^Confidence intervals and *P *values were derived using the bootstrap resampling method.

^c^*P *< 0.0001 for change in hospitalization.

CI = confidence interval; RLAT = risperidone long-acting therapy.

## Discussion

This analysis was conducted to examine the effectiveness of RLAT for patients with schizophrenia in real-world clinical settings in the US, Spain, Australia, and Belgium. The patients' overall clinical severity and functional levels at baseline were relatively similar across countries. Improvement in disease severity and functioning were seen in all patient groups and was maintained up to 24 months of follow-up. These results, consistent with those of other naturalistic studies with RLAT [22-24], further suggest that RLAT is effective across health care delivery systems.

The discontinuation rate associated with oral antipsychotics is known to be high [25]. In this analysis, completion rates over 24 months with RLAT ranged from 39.3% in the US to 62.7% in Spain. The variation in discontinuation rates among the four countries may be due to differences in access to treatment, social support (for example, in patients who live alone), and cost (for example, in Spain, RLAT is free for most patients).

RLAT was associated with decreased psychiatric hospitalization in all four countries, when patients were considered regardless of hospitalization status at baseline: hospitalizations per person-year decreased by approximately half in the year after initiation of RLAT, compared with the previous year. When the analysis was limited to outpatients at baseline, hospitalizations decreased significantly in the US and Spain. These data suggest the difficulties of assessing the treatment effect on inpatient status for patients with schizophrenia across varying health care systems. For example, 95% of patients in the US were outpatients at baseline, compared with only 48% in Australia. Beyond the individual patient factors that determine hospitalization status, such as disease severity, these results suggest major health care system difference regarding hospitalizations and duration of hospitalization. The intent of the analysis was to compare populations with a similar clinical status (outpatients at baseline); however, the high rates of baseline hospitalizations in Australia and Belgium more likely reflect differences in practice rather than clinical status across populations.

Randomized controlled studies provide the highest standard of evidence for the efficacy and safety of treatments; however, several disadvantages limit the generalizability of results. Most studies in schizophrenia are relatively short, even though patients require lifelong treatment. Further, because populations are small and limited by restrictive enrollment criteria, they may not be representative of clinical practice. Pragmatic studies, such as those described here, provide complementary data that evaluate whether an intervention works in real-life situations [26]. Pragmatic studies of patients with schizophrenia include a broader patient range, larger numbers of patients, and longer treatment durations and have provided insights into the effectiveness of antipsychotic treatment [27-30].

Several limitations should be noted. Because the eSTAR and SOURCE studies were not randomized, establishing causal relationships between RLAT and improvements in effectiveness measures is not possible. Further, patients with only a baseline visit were excluded from the analysis of hospitalization; however, this group comprised only a small subset of patients in the total sample (n = 168; 5.5%). Also, information on hospitalization in the 12 months before RLAT initiation was collected retrospectively. These data relied on the accuracy of historical chart information, and hospitalization might have been underreported. Data were analyzed only for patients who completed 24 months of treatment and who had a baseline and a 24-month value on the CGI-S and GAF scales, which may have introduced selection bias. Improvements in effectiveness measures were not statistically analyzed for differences between the participating countries because certain factors that might not be properly adjusted or controlled might contribute to country differences.

## Conclusions

Pragmatic studies are valuable for capturing real-world clinical practices across different health care settings and can be used to inform health care decision makers. Despite substantial variability among health care systems, treatment with RLAT resulted in improved functioning and decreased psychiatric hospitalization rates in patients with schizophrenia from the US, Spain, Australia, and Belgium.

## Competing interests

TL is a consultant for Janssen, Eli Lilly, and Pfizer; has received grant-research support from Janssen; and is a member of a speaker bureau for Janssen, Eli Lilly, Pfizer, and AstraZeneca. JMO is a member of regional, national, and international advisory boards for Janssen-Cilag, Eli Lilly, AstraZeneca, and Bristol-Myers Squibb; is involved in designing and participating in clinical trials for Janssen-Cilag, Eli Lilly, AstraZeneca, Pfizer, Lundbeck, GlaxoSmithKline, and Bristol-Myers Squibb; and has received educational grants for research, honoraria, and travel support for activities as a consultant/advisor and lecturer/faculty member for Janssen-Cilag, Eli Lilly, AstraZeneca, Pfizer, Lundbeck, GlaxoSmithKline, Novartis, and Bristol-Myers Squibb. JP is a consultant and member of the speaker bureaus for and received grant-research support from Janssen-Cilag, Eli Lilly, AstraZeneca, Lundbeck, and Bristol-Myers Squibb. CMK is a consultant for Ortho-McNeil Janssen Scientific Affairs, LLC. CC, SCR, and RD are employees of Ortho-McNeil Janssen Scientific Affairs, LLC, and Johnson & Johnson stockholders. WM was an employee of Ortho-McNeil Janssen Scientific Affairs LLC at the time of this analysis. LM is an employee of Johnson & Johnson Pharmaceutical Research and Development, LLC, and a Johnson & Johnson stockholder. AJ is an employee of Johnson & Johnson Pharmaceutical Services and a Johnson & Johnson stockholder. KSA was an employee of Johnson & Johnson Pharmaceutical Services at the time of this analysis. CD and PO have no competing interests.

## Authors' contributions

TL, JMO, JP, and CD were involved in the interpretation of data, and critical drafting and revising of the manuscript for important intellectual content. PO, CMK, CC, SCR, RD, WM, LM, AJ, and KSA contributed to the conception and design, acquisition of data, analysis and interpretation of data, and drafting of the manuscript and its critical revision for important intellectual content.

## References

[B1] World Health Organization World Mental Health Atlas 2005 Geneva, Switzerland: WHO

[B2] Eli Lilly and Company Keeping care complete: a caregivers' perspective on mental illness and wellness. An international survey 2010 Indianapolis, IN: Eli Lilly and Company

[B3] Eli Lilly and Company Keeping care complete: a physicians' perspective on mental illness and wellness. An international survey: International findings sheet 2010 Indianapolis, IN: Eli Lilly and Company

[B4] Zhu B Ascher-Svanum H Faries DE Peng X Salkever D Slade EP Costs of treating patients with schizophrenia who have illness-related crisis events BMC Psychiatry 2008 8 72 10.1186/1471-244X-8-72 18727831 PMC2533651

[B5] Lacro JP Dunn LB Dolder CR Leckband SG Jeste DV Prevalence of and risk factors for medication nonadherence in patients with schizophrenia: a comprehensive review of recent literature J Clin Psychiatry 2002 63 892 909 10.4088/JCP.v63n1007 12416599

[B6] Docherty JP Grogg AL Kozma C Mahmoud R Antipsychotic maintenance in schizophrenia: partial compliance and clinical outcome Presented at The American College of Neuropsychopharmacology 41st Annual Meeting 2002 San Juan, Puerto Rico

[B7] Gilmer TP Dolder CR Lacro JP Folsom DP Lindamer L Garcia P Jeste DV Adherence to treatment with antipsychotic medication and health care costs among Medicaid beneficiaries with schizophrenia Am J Psychiatry 2004 161 692 699 10.1176/appi.ajp.161.4.692 15056516

[B8] Valenstein M Copeland LA Blow FC McCarthy JF Zeber JE Gillon L Bingham CR Stavenger T Pharmacy data identify poorly adherent patients with schizophrenia at increased risk for admission Med Care 2002 40 630 639 10.1097/00005650-200208000-00002 12187177

[B9] Weiden PJ Kozma C Grogg A Locklear J Partial compliance and risk of rehospitalization among California Medicaid patients with schizophrenia Psychiatr Serv 2004 55 886 891 10.1176/appi.ps.55.8.886 15292538

[B10] Ascher-Svanum H Faries DE Zhu B Ernst FR Swartz MS Swanson JW Medication adherence and long-term functional outcomes in the treatment of schizophrenia in usual care J Clin Psychiatry 2006 67 453 460 10.4088/JCP.v67n0317 16649833

[B11] Sun SX Liu GG Christensen DB Fu AZ Review and analysis of hospitalization costs associated with antipsychotic nonadherence in the treatment of schizophrenia in the United States Curr Med Res Opin 2007 23 2305 2312 10.1185/030079907X226050 17697454

[B12] Lieberman JA Sheitman BB Kinon BJ Neurochemical sensitization in the pathophysiology of schizophrenia: deficits and dysfunction in neuronal regulation and plasticity Neuropsychopharmacology 1997 17 205 229 10.1016/S0893-133X(97)00045-6 9326746

[B13] Kane JM Aguglia E Altamura AC Ayuso Gutierrez JL Brunello N Fleischhacker WW Gaebel W Gerlach J Guelfi JD Kissling W Lapierre YD Lindstrøm E Mendlewicz J Racagni G Carulla LS Schooler NR Guidelines for depot antipsychotic treatment in schizophrenia. European Neuropsychopharmacology Consensus Conference in Siena, Italy Eur Neuropsychopharmacol 1998 8 55 66 10.1016/S0924-977X(97)00045-X 9452941

[B14] Schooler NR Relapse and rehospitalization: comparing oral and depot antipsychotics J Clin Psychiatry 2003 64 14 17 10.4088/JCP.v64n0105 14680414

[B15] Kane JM Eerdekens M Lindenmayer JP Keith SJ Lesem M Karcher K Long-acting injectable risperidone: efficacy and safety of the first long-acting atypical antipsychotic Am J Psychiatry 2003 160 1125 1132 10.1176/appi.ajp.160.6.1125 12777271

[B16] Chue P Eerdekens M Augustyns I Lachaux B Molcan P Eriksson L Pretorius H David AS Comparative efficacy and safety of long-acting risperidone and risperidone oral tablets Eur Neuropsychopharmacol 2005 15 111 117 10.1016/j.euroneuro.2004.07.003 15572280

[B17] Simpson GM Mahmoud RA Lasser RA Kujawa M Bossie CA Turkoz I Rodriguez S Gharabawi GM A 1-year double-blind study of 2 doses of long-acting risperidone in stable patients with schizophrenia or schizoaffective disorder J Clin Psychiatry 2006 67 1194 1203 10.4088/JCP.v67n0804 16965196

[B18] Fleischhacker WW Eerdekens M Karcher K Remington G Llorca PM Chrzanowski W Martin S Gefvert O Treatment of schizophrenia with long-acting injectable risperidone: a 12-month open-label trial of the first long-acting second-generation antipsychotic J Clin Psychiatry 2003 64 1250 1257 10.4088/JCP.v64n1017 14658976

[B19] Chue P Llorca PM Duchesne I Leal A Rosillon D Mehnert A Hospitalization rates in patients during long-term treatment with long-acting risperidone injection J Appl Res 2005 5 266 274

[B20] Guy W (Ed) ECDEU Assessment Manual for Psychopharmacology 1976 Rockville, MD: US Dept of Health Education and Welfare, National Institute of Mental Health 218 222

[B21] Jones SH Thornicroft G Coffey M Dunn G A brief mental health outcome scale-reliability and validity of the Global Assessment of Functioning (GAF) Br J Psychiatry 1995 166 654 659 10.1192/bjp.166.5.654 7620753

[B22] Taylor DM Young CL Mace S Patel MX Early clinical experience with risperidone long-acting injection: a prospective, 6-month follow-up of 100 patients J Clin Psychiatry 2004 65 1076 1083 10.4088/JCP.v65n0808 15323592

[B23] De Marinis T Saleem PT Glue P Arnoldussen WJ Teijeiro R Lex A Latif MA Medori R Switching to long-acting injectable risperidone is beneficial with regard to clinical outcomes, regardless of previous conventional medication in patients with schizophrenia Pharmacopsychiatry 2007 40 257 263 10.1055/s-2007-992140 18030649

[B24] Niaz OS Haddad PM Thirty-five months experience of risperidone long-acting injection in a UK psychiatric service including a mirror-image analysis of in-patient care Acta Psychiatr Scand 2007 116 36 46 10.1111/j.1600-0447.2006.00980.x 17559599

[B25] Lieberman JA Stroup TS McEvoy JP Swartz MS Rosenheck RA Perkins DO Keefe RS Davis SM Davis CE Lebowitz BD Severe J Hsiao JK Clinical Antipsychotic Trials of Intervention Effectiveness (CATIE) Investigators Effectiveness of antipsychotic drugs in patients with chronic schizophrenia N Engl J Med 2005 353 1209 1223 10.1056/NEJMoa051688 16172203

[B26] Benson K Hartz AJ A comparison of observational studies and randomized, controlled trials N Engl J Med 2000 342 1878 1886 10.1056/NEJM200006223422506 10861324

[B27] Haro JM Edgell ET Novick D Alonso J Kennedy L Jones PB Ratcliffe M Breier A Effectiveness of antipsychotic treatment for schizophrenia: 6-month results of the Pan-European Schizophrenia Outpatient Health Outcomes (SOHO) study Acta Psychiatr Scand 2005 111 220 231 10.1111/j.1600-0447.2004.00450.x 15701107

[B28] Moller HJ Llorca PM Sacchetti E Martin SD Medori R Parellada E Efficacy and safety of direct transition to risperidone long-acting injectable in patients treated with various antipsychotic therapies Int Clin Psychopharmacol 2005 20 121 130 10.1097/00004850-200505000-00001 15812261

[B29] Jones PB Barnes TRE Davies L Dunn G Lloyd H Hayhurst KP Murray RM Markwick A Lewis SW Randomized controlled trial of the effect on Quality of Life of second- vs first-generation antipsychotic drugs in schizophrenia: Cost Utility of the Latest Antipsychotic Drugs in Schizophrenia Study (CUtLASS 1) Arch Gen Psychiatry 2006 63 1079 1087 10.1001/archpsyc.63.10.1079 17015810

[B30] Tandon R Marcus RN Stock EG Riera LC Kostic D Pans M McQuade RD Nyilas M Iwamoto T Crandall DT A prospective, multicenter, randomized, parallel-group, open-label study of aripiprazole in the management of patients with schizophrenia or schizoaffective disorder in general psychiatric practice: Broad Effectiveness Trial With Aripiprazole (BETA) Schizophr Res 2006 84 77 89 10.1016/j.schres.2005.12.857 16483745

